# Local Delivery of Polarized Macrophages Improves Reperfusion Recovery in a Mouse Hind Limb Ischemia Model

**DOI:** 10.1371/journal.pone.0068811

**Published:** 2013-07-24

**Authors:** Nadine Jetten, Marjo M. P. C. Donners, Allard Wagenaar, Jack P. M. Cleutjens, Nico van Rooijen, Menno P. J. de Winther, Mark J. Post

**Affiliations:** 1 Department of Molecular Genetics, Cardiovascular Research Institute Maastricht (CARIM), Maastricht, The Netherlands; 2 Department of Physiology, Cardiovascular Research Institute Maastricht (CARIM), Maastricht, The Netherlands; 3 Department of Pathology, Cardiovascular Research Institute Maastricht (CARIM), Maastricht, The Netherlands; 4 Department of Medical Biochemistry, Academic Medical Center (AMC), Amsterdam, The Netherlands; 5 Department of Molecular Cell Biology, Faculty of Medicine, Vrije Universiteit (VU), Amsterdam, The Netherlands; University Heart Centre Freiburg, Germany

## Abstract

**Aims:**

Enhancement of collateral development in coronary or peripheral artery disease is a therapeutic target, but it has proven difficult to achieve. Macrophages are key players in collateral remodeling, yet the effect of different macrophage subsets on arteriogenesis has not been investigated.

**Methods and Results:**

Murine macrophages were cultured from bone marrow and polarized into M1 (IFNγ), M2a (IL-4) or M2c (IL-10) subsets. C57BL/6 mice underwent femoral artery ligation followed by intramuscular injection of macrophage subsets. Using eGFP expressing macrophages, cells could be detected at least 6 days after ligation and were located in the perivascular space of collateral vessels. After 14 days, perfusion ratio was increased in animals treated with M1 as well as M2a and M2c macrophages compared to control. Depletion of circulating monocytes by clodronate liposome injections did not hamper reperfusion recovery, however, treatment with exogenous polarized macrophages improved perfusion ratio after 14 days again. We used IL10R^fl/fl^/LysMCre^+^ mice to study the effect of inhibition of endogenous polarization towards specifically M2c macrophages on arteriogenesis. Deletion of the IL10-receptor (IL10R) in the myeloid lineage did not affect reperfusion recovery, yet the pro-arteriogenic effect of exogenously injected M2c macrophages was still present.

**Conclusions:**

Local injection of polarized macrophages promotes reperfusion recovery after femoral artery ligation and is not influenced by depletion of circulatory monocytes. Preventing endogenous M2c polarization did not affect reperfusion recovery suggesting that M2c’s are not required for collateralization, but are sufficient to induce collateral formation upon exogenous administration. This is the first study using local injection of macrophage subsets showing the pro-arteriogenic effect of polarized macrophages.

## Introduction

Many people worldwide suffer from the consequences of arterial occlusion common for coronary artery disease or peripheral artery disease (CAD and PAD). Drug therapies have been designed to restore blood flow artificially, but until now without robust clinical results [1]. Stimulating natural reperfusion recovery by promoting the transformation of pre-existing arterioles into larger conductance arteries, also termed arteriogenesis, is a very attractive target for designing new treatment strategies.

Acute occlusion of a major conductance artery, due to plaque rupture and thrombosis formation, decreases the blood perfusion rate dramatically and will eventually lead to post-occlusive ischemic tissues. Consequently, a steep pressure gradient develops over pre-existing collateral anastomoses leading to an increased fluid shear stress, which activates the endothelium and triggers the initiation of the remodeling process [2,3]. Decades ago, it was already demonstrated that collateral growth is highly associated with macrophage accumulation around the collateral vasculature [4]. Monocytes from the circulation adhere to the activated endothelium, expressing adhesion molecules (ICAM-1 [5], VCAM-1) and MCP-1 [6], and subsequently transmigrate into the perivascular tissue where they mature into macrophages [7]. Being a rich source of cytokines and growth factors, these macrophages can stimulate the proliferation of endothelial cells and smooth muscle cells [8]. In addition, the prototypic monocyte recruiting cytokines, MCP-1 [9] and GM-CSF, increase collateral formation on their own but also synergistically by prolonging life of monocytes and residence time of macrophages [10].

The importance of monocytes and macrophages on the progression of arteriogenesis was demonstrated by using monocyte-deficient op/op mice [11]. Femoral artery ligation resulted in more than 40% impaired reperfusion recovery and in a significant decrease in collateral arteries. Likewise, depleting circulating monocytes by 5-fluorouracil (5-FU) treatment attenuated flow recovery, which could be rescued by intravenous injections of blood-isolated monocytes [12]. Finally, when monocyte recruitment was inhibited by knocking out the MCP-1 receptor (CCR2), flow recovery was also significantly decreased [13].

For several years now it is known that different subsets of macrophages coexist *in vivo* and exert different functions depending on their environmental stimulus. We define pro-inflammatory M1 macrophages (induced by LPS and/or IFNγ) and anti-inflammatory M2 macrophages (induced by IL-4/IL-13 or IL10) [14,15]. M2 macrophages are pro-angiogenic as was already demonstrated in vitro [16] and in vivo by our previous data, indicating a pro-angiogenic effect of M2 macrophages by increased production of FGF-2 and PlGF (manuscript submitted). Since angiogenic factors are considered to stimulate arteriogenesis [17], we hypothesize that M2 macrophages also have pro-arteriogenic capacities. Though some knowledge on macrophage subsets in relation to arteriogenesis is gained [18], intervention studies on this particular process have not been performed.

In this study we investigated the effect of macrophage subsets on reperfusion recovery and collateral remodeling *in vivo*. We demonstrated for the first time that local injection of polarized macrophages improves reperfusion recovery regardless of the subset. Polarization of macrophages therefore is important for collateral formation, but the effect is independent of the direction of polarization.

## Methods

### Ethics statement

Animals were held under the guidelines of the animal care facility (Maastricht University) with unlimited access to food and drinking water. All animal experiments were approved by the Committee for Animal Welfare of the Maastricht University and conforms the Directive 2010/63/EU of the European Parliament.

### Bone marrow isolation

Bone marrow was isolated from the femur and tibia of C57BL/6 mice 10-14 weeks of age as described previously [19]. Cells were differentiated into bone marrow derived macrophages by culture in R10 medium for 8 days (RPMI-1640 medium (Gibco Invitrogen, Breda, The Netherlands), 10% heat-inactivated Fetal Calf Serum (FCS) (Bodinko BV, Alkmaar, The Netherlands), L-glutamine 2mM, 100 U/ml penicillin, 100µg/ml streptomycin and 10mM HEPES (all Gibco Invitrogen, Breda, The Netherlands)) supplemented with 15% L929-conditioned medium (LCM) containing M-CSF.

### Macrophage polarization

After 8 days, differentiated macrophages were harvested, counted and suspended in R10 + 15% LCM. Cells (3.5x10^5^ cells/well) were plated in 24-wells plates and kept overnight in order to adhere again. The next day, differentiated macrophages were polarized for 24h with interferon-γ (IFNγ) (100U/ml, Hycult biotech) for M1, interleukin (IL)-4 (20ng/ml, Peprotech) for M2a or IL-10 (10ng/ml, R&D Systems) for M2c macrophages. Non-polarized cells are referred to as M0 macrophages.

### Animal models

Forty-five (n=9/group) female C57Bl/6 mice (10-12 weeks old, Charles River, France) underwent surgical procedures to induce hind limb ischemia (as described below). Directly after ligation, animals were treated with NaCl or cell suspension containing macrophage subsets (M0, M1, M2a or M2c) and by Laser Doppler Imaging the reperfusion recovery was measured over time.

#### Clodronate liposomes

For a macrophage depletion experiment, 45 female C57Bl/6 mice (10-12 weeks old, Charles River, France) were subjected to i.v. injection of clodronate loaded liposomes (200µl) (Roche, Mannheim, Germany) into the lateral tail vein [20]. First injection was provided 24h prior to femoral artery ligation followed by a second injection (50µl) post-operatively.

#### eGFP macrophages

Bone marrow of RA/EG mice (n=4), kindly provided by C. van’t Veer (Academic Medical Center, Amsterdam), was isolated and differentiated into bone marrow derived macrophages. Cells of the myeloid lineage of these mice express enhanced green fluorescent protein (eGFP) [21]. Forty female C57Bl/6 mice (10-12 weeks old, Charles River, France) underwent femoral artery ligation, followed by injection of eGFP macrophage subsets (M0, M1, M2a or M2c). Animals were subsequently sacrificed at day 2 (n=6), 4 (n=2) or 6 (n=2) after surgery for histological analysis.

#### IL10R1^fl/fl^/LysMCre mice

Femoral arteries of 27 IL10R1^fl/fl^/LysMCre mice [22] (n=9, IL10R1^fl/fl^/LysMCre^-^; n=18, IL10R1^fl/fl^/LysMCre^+^) were ligated and reperfusion recovery was measured. These mice have a myeloid cell specific deficiency of the interleukin-10 receptor-1 (IL10R1), leading to strongly reduced sensitivity of these cells to IL-10 [23] and subsequently lack of in vivo IL-10 induced M2c macrophages. One group (n=9, IL10R1^fl/fl^/LysMCre^+^) was treated with exogenous M2c macrophages directly after the surgical procedure.

### Femoral artery ligation

Surgical procedures to induce hind limb ischemia were performed as described before [24]. During the whole procedure mice were anaesthetized with isoflurane (1.5-2%) and sedated 30 minutes before and 1 hour after the procedure with Temgesic (0.5 mg/BW). Ligations were placed at the site of bifurcation of the femoral artery and the epigastric artery, on the epigastric artery itself and at the bifurcation of the femoral artery and the popliteal artery. Directly after ligation, NaCl or cell suspension of macrophage subsets was injected via the open wound into the adductor muscle. In the region between the profunda artery, the saphenous artery and the femoral artery, 5x10^5^ cells (total volume of 60µl) were injected divided over three injection sites in the area where collateral vessels are located.

### Laser Doppler Imaging

Under standardized conditions, blood flow in the hind legs of all animals was measured using Laser Doppler Imaging (LDI) (Perimed, Sweden). Measurements were performed before and directly after the surgical procedure and at post-operative days 3, 7 and 14. During the measurements mice were placed on a heating pad in a climate controlled chamber (37^°^C) to ensure minimal variation between flow measurements. The perfusion ratio per time point was calculated by dividing the perfusion of the ligated by the non-ligated leg.

### FACS

Blood was collected at day 1 and 3 after PBS- or clodronate-liposome injection and analyzed with flow cytometry to study the effect of clodronate treatment on blood leukocyte populations. After lysis of red blood cells, leukocytes were stained with antibodies against CD3 (Ebioscience, clone 145-C11), CD45R/B220 (Ebioscience, clone RA3-6B2), Ly6G (BD, clone 1A8) and CD11b (BD, clone M1/70) to discriminate between T-cells (CD3^+^ B220^-^), B-cells (CD3^-^ B220^+^), granulocytes (CD3^-^ B220^-^ Ly6G^+^ CD11b^+^) and monocytes (CD3^-^ B220^-^ Ly6G^-^ CD11b^+^) respectively. Leukocyte populations were measured using a FACS CANTO II and data were analyzed by using the FACSDiva software (BD Biosciences, CA, USA).

### Immunohistochemistry

Tissues were prepared for immunohistochemical analysis and stained for endothelial marker CD31 (PECAM-1) (BD biosciences). Adductor muscles were isolated and embedded in O.C.T. Tissue Tek (Sakura Finetek), snap-frozen in isopentane and stored at -80°C. Frozen sections (7µm) were fixed in acetone, air dried and rehydrated in PBS. Sections were pre-incubated with a biotin blocking system (VectorLabs) to prevent non-specific binding. After incubation with the primary (CD31, [1:200]) and secondary mouse-anti-rat antibody (Dako), staining was amplified by incubating sections with ABC reagent (VectorLabs). Sections were developed using an AEC substrate kit (VectorLabs) for 15 minutes followed by hematoxylin counterstaining. Analysis of vessel number/mm^2^ and size was performed using a morphometry program Qwin version 3.5.1 (Leica, Cambridge, UK), where after vessels were categorized according to their diameter.

Sections of adductor muscles of eGFP-macrophage injected animals were stained with a monoclonal antibody against GFP (Invitrogen). Sections were fixed in acetone supplemented with 0.5% H_2_O_2_, air dried and rehydrated in PBS. Anti-GFP [1:50] was incubated overnight with blocking fetal calf serum (FCS). Bright vision (Immunologic) was used to amplify the staining and sections were developed using AEC substrate for 15 minutes followed by hematoxylin counterstaining.

### Statistical analysis

All data are presented as mean ± SEM. Laser Doppler measurements were analyzed using SPSS 17.0 repeated measures ANOVA and LSD post-hoc test. In all cases, p-value ≤0.05 was considered to be significant.

## Results

### Exogenously injected macrophages remain located in the muscle up to 6 days after ligation

**Figure 1:**
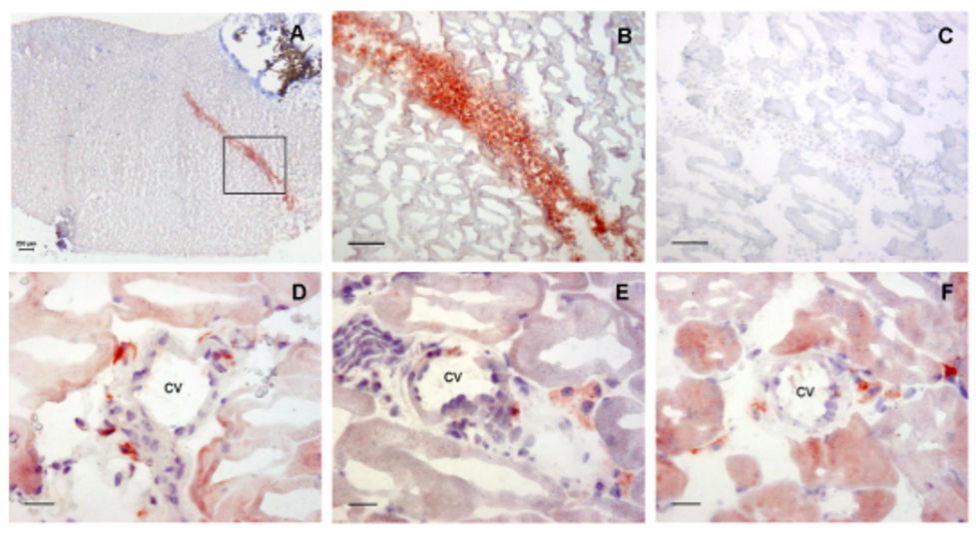
Visualization of eGFP-macrophages in muscle tissue (n=4). A, Overview of muscle tissue after ligation and macrophage injection. B, eGFP-macrophages located in the injection site at post-operative day 2 (scale bar = 100 µm). C, Negative control eGFP staining. Injected macrophages could be detected in the perivascular space of collateral vessels at day 2 (D), day 4 (E) and day 6 (F) (scale bar = 20µm).

Localization of exogenous macrophages during the reperfusion recovery period was visualized using eGFP expressing macrophages in a hind limb ischemia model. Directly after femoral artery ligation mice were injected with eGFP-macrophages and sacrificed at day 2, 4 or 6. After 2 days, injected eGFP-macrophages could be clearly visualized within the adductor muscle. Overall, cells are clustered at the injection site in between the muscle fibers (Figure 1A–C). Already at this time point some eGFP-macrophages were located in the perivascular space of collateral vessels (Figure 1D). Moreover, injected macrophages were found in a more dispersed distribution throughout the muscle after 4 and 6 days after ligation. Cells migrated out of the injection site towards the site of ligation and most interestingly towards the perivascular space of collateral vessels (Figure 1E,F).

### Treatment with polarized macrophages results in improvement of reperfusion recovery

**Figure 2:**
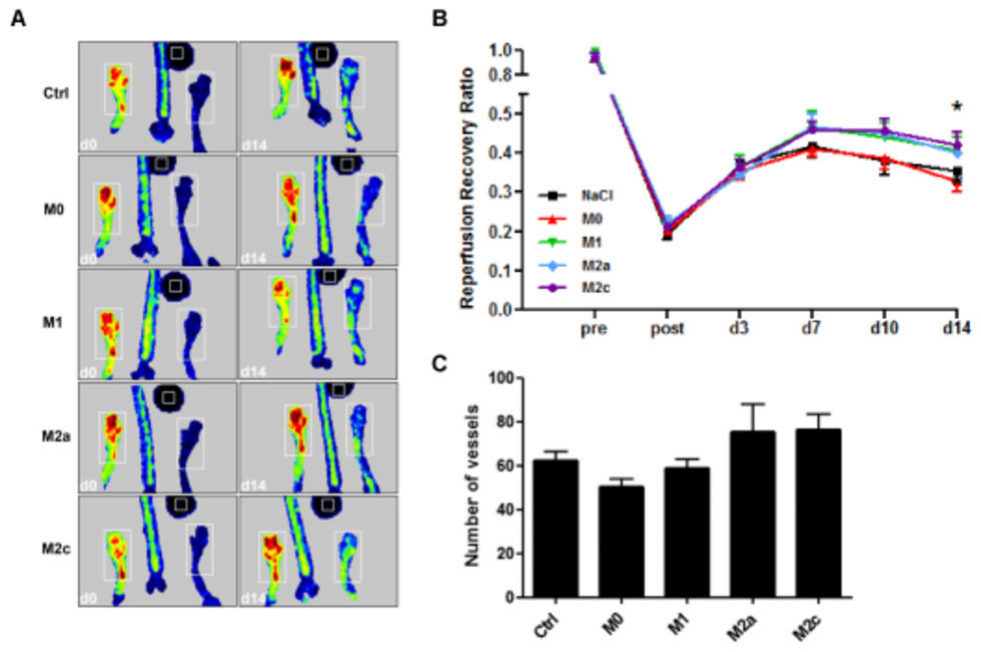
Reperfusion recovery after femoral artery ligation and macrophage treatment (n=9). A, Representative Laser Doppler images of each group post-operatively and at day 14. B, Treatment with M1, M2a and M2c macrophages increases the reperfusion recovery after 14 days. C, A trend towards an increase in vessel number could be detected in the category ’50-60 µm diameter’ in animals treated with M2a and M2c macrophages.

Our previous data show that M2 macrophages increase angiogenesis in vitro and in vivo by inducing the expression of the pro-angiogenic growth factors FGF-2 and PlGF (manuscript submitted). Arteriogenesis however is a distinct mode of neovascularization and is triggered by hemodynamic stimuli rather than by ischemia. Since angiogenesis and arteriogenesis are considered to encompass similar mechanisms, we examined the effect of different macrophage subsets on arteriogenesis by local injection of M0, M1, M2a and M2c macrophages i.m. directly after femoral artery ligation and measured reperfusion recovery. As a control for cell injections we injected the same volume of NaCl into the muscle. By means of LDI the reperfusion recovery over time was measured and perfusion ratio (non-ligated/ligated) was analyzed (Figure 2A). Directly after the ligation procedure the average perfusion ratio dropped to ~20% of baseline. The initial reperfusion recovery did not differ between groups and reached an average ratio of 36.5% ± 0.01 3 days after the operation procedure. After 14 days, we observed a trend in reperfusion recovery in animals treated with the M1 (40.7% ± 0.1, p = 0.07) and the M2a subset (39.9% ± 0.1, p = 0.07) and a significant improvement in animals treated with the M2c subset (42.1% ± 0.1, p = 0.05) compared to treatment with M0 macrophages (Figure 2B). The perfusion ratio levels of NaCl and M0 treated animals did not rise above 37.3% ± 0.1 and 32.8% ± 0.1 respectively. After quantitative immunohistochemical analysis we could detect a trend towards an increase in number of vessels of 50-60 µm diameter in the M2a and M2c treated animals.

### Polarized exogenous macrophages improve reperfusion recovery regardless of endogenous macrophages depletion

**Figure 3:**
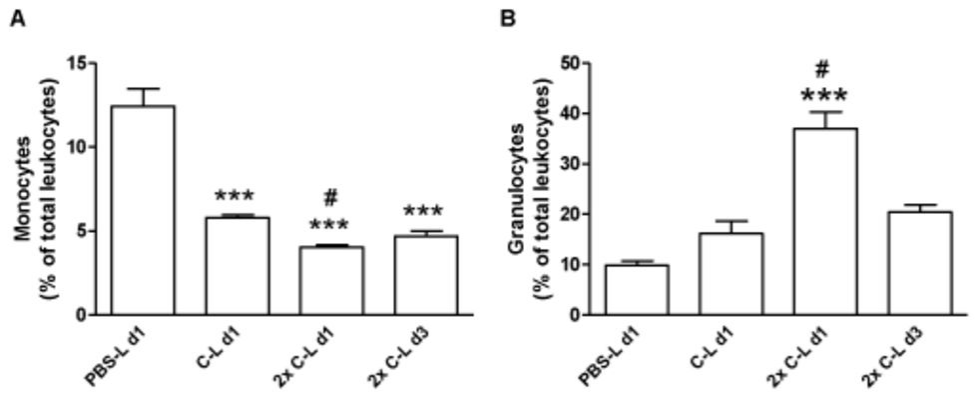
Effect of chlodronate liposomes on blood monocyte and granulocyte population (n=6). Clodronate liposome treatment reduces the endogenous monocyte population significantly compared to PBS liposome treatment (***: p<0.001) and compared to single clodronate injection (#: p<0.05). A, Single injection with clodronate reduces the monocyte population with 53%, whereas a second injection results in 68% reduction. B, Granulocyte population increases upon clodronate treatment after double injections with clodronate compared to PBS-liposome treatment (***: p<0.001) and single clodronate injection (#: p<0.001), most probably due the depletion of the monocyte population.

To study the contribution of the endogenous macrophage population on effect of polarized macrophages we repeated the treatment experiments after pretreatment with clodronate liposomes. After a one-time injection of clodronate liposomes we observed a reduction in the blood monocyte population of 53% ± 0.35(p<0.001) compared to injection with PBS loaded liposomes (Figure 3A). When treated a second time with clodronate directly after the surgical procedure we observed a reduction of 68% ± 0.59(p<0.001) that was sustained for at least three days (62% ± 1.29; p<0.001) after surgery. Relative increase (60%) in the granulocyte population in response to the clodronate liposome treatment could be observed which was increased even more in response to the second injection (129%) (Figure 3B).

**Figure 4:**
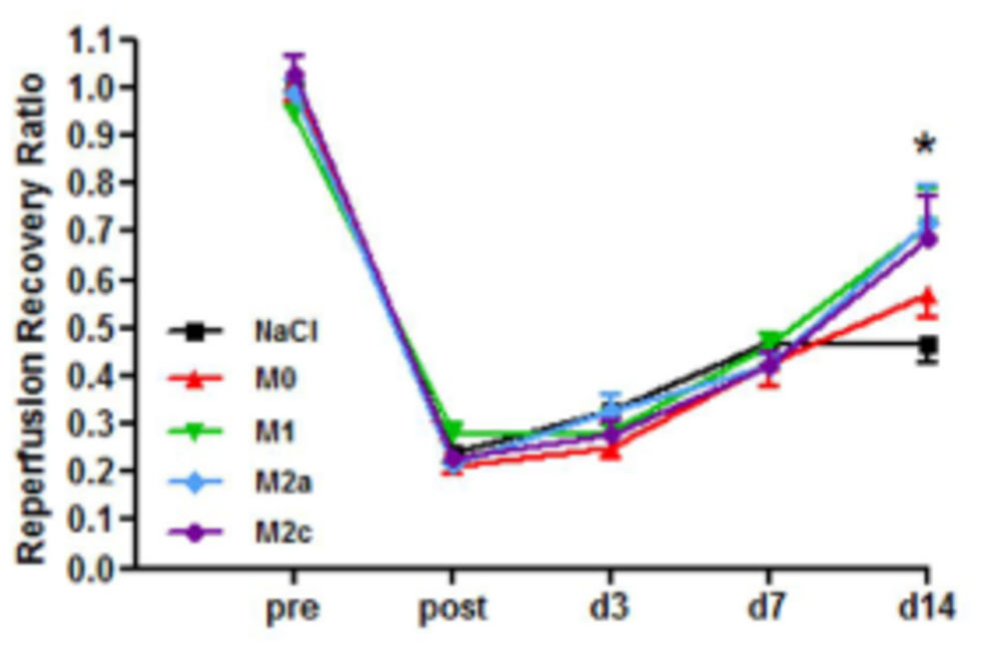
Reperfusion recovery after clodronate liposome treatment (n=9). Reperfusion recovery is increased at day 14 in animals treated with either M1, M2a or M2c macrophages compared to NaCl treatment (***: p<0.001). Depletion of the circulating monocytes/macrophages did not inhibit reperfusion recovery and did not alter the effect of exogenously injected polarized macrophages.

Surprisingly, reperfusion recovery was not impaired by clodronate liposome injection when compared to animals that underwent the same procedure without the clodronate treatment up to day 7, suggesting that depletion of endogenous macrophages does not affect collateral remodeling. No differences in vessel number and diameter could be detected. However, consistent with our previous results we observed an increased perfusion ratio after 14 days in mice treated with M1 (71.5% ± 0.2, p < 0.001), M2a (71.8% ± 0.2, p < 0.001) or M2c macrophages (68.3% ± 0.2, p < 0.001) compared to M0 (56.8% ± 0.1) and NaCl treatment (46.3% ± 0.1) (Figure 4).

In conclusion, administration of exogenously polarized macrophages improves reperfusion recovery after induction of hind limb ischemia, irrespective of their polarization status and regardless of depletion of the endogenous macrophage pool.

### Exogenously, not endogenously polarized macrophages improve reperfusion recovery

**Figure 5:**
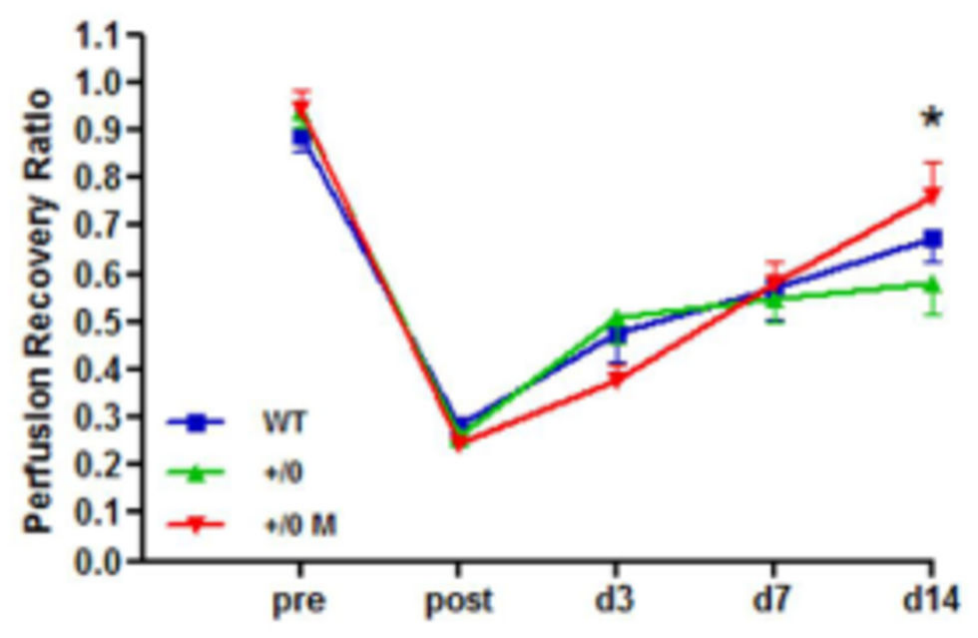
Effect of inhibiting endogenous macrophage polarization (n=9). Reperfusion recovery in IL10R1^fl/fl^/LysMCre- (WT) and IL10R1^fl/fl^/LysMCre+ (KO) mice. Treatment with M2c macrophages improves reperfusion recovery in IL10R1^fl/fl^/LysMCre+ mice after 14 days compared to untreated animals (*: p<0.05).

In order to study the effect of endogenously polarized M2c macrophages we used mice with a conditional deletion for the IL10 receptor (IL10R1^fl/fl^/LysMCre^+^) on myeloid cells. Consequently, macrophages of these mice are not responsive to IL10 and cannot polarize towards an M2c phenotype. After 14 days, a slight delay in reperfusion recovery could be observed in IL10R1^fl/fl^/LysMCre^+^ (57.9% ± 0.195) compared to IL10R1^fl/fl^/LysMCre^-^ animals (67.5% ± 0.146), although the effect was not significant. When exogenous M2c macrophages were injected into the adductor muscle of IL10R1^fl/fl^/LysMCre^+^ mice, reperfusion recovery improved significantly (76.2% ± 0.217, p<0.05) compared to the IL10R1^fl/fl^/LysMCre^+^ without macrophage treatment (Figure 5). This indicates that reperfusion recovery is not dependent on endogenous polarization of M2c macrophages and that treatment with exogenously polarized cells positively influences arteriogenesis.

## Discussion

In this study we investigated the effect of exogenous macrophage subsets on reperfusion recovery and collateral remodeling *in vivo*. We showed that local injection of exogenously polarized macrophages of the M1 as well as the M2 subset improves reperfusion recovery. The effect was not ablated by depletion of endogenous circulating monocytes and macrophages by clodronate liposome treatment, which emphasizes the pro-arteriogenic effect of the exogenous cells. M2c macrophages are not required for arteriogenesis as the IL10R1^fl/fl^/LysMCre^+^ myeloid cells allowed normal reperfusion recovery. In the IL10R1^fl/fl^/LysMCre^+^ mice however, exogenously administered M2c macrophages were still able to induce arteriogenesis. Polarization of macrophages therefore is sufficient to induce collateral formation and it seems independent on the direction of polarization.

The effect of macrophage heterogeneity on arteriogenesis was investigated in a study performed by Takeda et al [18]. Contrary to our own study in which we demonstrated the effect of local administration of exogenously polarized M1 and M2 macrophage subsets, they showed improved arteriogenesis after endogenous M2-like polarization by haplodeficiency of the oxygen sensor Phd2. Macrophages were characterized by increased gene expression levels of different M2 markers, but mainly arginase-1, which resembles an IL-4 induced polarization as was described by Loke et al. [25]. We showed that endogenous polarization of macrophages specifically into the M2c subset using the IL10R1^fl/fl^/LysMCre^+^ mice did not improve reperfusion recovery. Though endogenous IL-10 may be essential for reperfusion recovery, it probably does not rely solely on responsiveness of myeloid cells but also of endothelial cells and SMC.

Schaper et al demonstrated that macrophage accumulation in the vessel wall is correlated with increased collateral remodeling [4]. Since macrophage accumulation is reported to be maximal 3 days after femoral artery ligation, restraining the endogenous monocyte population in this critical phase should increase the opportunity for the exogenous cells to exert their function. To induce a sustained suppression of monocytes during the first three days after ligation multiple doses of clodronate liposomes were administered [26,27]. Although the monocyte population was severely reduced during this period, we did not observe an effect on reperfusion recovery. It was described that by i.v. injection of clodronate liposomes only circulating cells are depleted [20], leaving the tissue resident macrophages unaffected which could still support collateral remodeling. Khmelewski et al showed that depletion of bone marrow cells neither affects collateral growth nor macrophage accumulation, drawing the conclusion that in the early phase of collateral proliferation resident macrophages, rather than circulating monocytes, are essential [28]. We observed a large increase of the granulocyte population upon multiple clodronate injections, a phenomenon which was also reported in other depletion studies [11,12]. Hoefer et al investigated the effect of different leukocyte populations on arteriogenesis and concluded that attraction of leukocyte populations other than monocytes failed to induce arteriogenesis directly [29].

Our results on the stimulatory effect of locally injected cells were supported by a study in which marrow-derived stromal cells (MSC) were injected in the adductor muscle 24h after femoral artery ligation [30]. Treatment with the MSCs resulted in enhanced reperfusion recovery and improvement of collateral remodeling and limb function without incorporation of these cells into collateral vessels. It was already described that bone-marrow cells do not incorporate into the growing vasculature, but function as supporting cells [31]. This suggests a paracrine effect, which was demonstrated by increased expression of FGF-2 and VEGF in MSC treated limbs. Furthermore, implantation of a combination of peripheral blood mononuclear cells (PBMNCs) and platelets, both being producers of angiogenic growth factors, into the thigh muscle was successful in stimulating vessel formation and collateral remodeling [32]. These studies confirm our findings on the improvement of arteriogenesis by local administration of exogenous cells. By injection of eGFP expressing macrophages we could localize exogenously injected macrophages for at least 6 days after surgical procedure. Moreover, we could observe their presence in the perivascular space of collateral vessels at different time points. Herewith, we validate the prevalence of macrophages during the early phase of arteriogenesis that might be recruited from local sites rather than from the circulation.

Unexpectedly, treatment with M1 macrophages resulted in equal improvement in blood perfusion as treatment with M2a or M2c macrophages. Arras et al. already showed that accumulated monocytes around collateral vessels are the main producers of TNFα and FGF-2 [31]. By double staining, they nicely showed co-localization of the rabbit macrophage marker RAM-11 and either FGF-2 or TNFα expression. Pro-angiogenic cytokines and growth factors also share pro-arteriogenic properties. The pro-angiogenic effect of TNFα produced by M1 macrophage was demonstrated *in vitro* [33]. In rabbits, the effect of TNFα on arteriogenesis was investigated by treating animals with different TNFα antagonists after femoral artery occlusion. Here, both collateral growth and conductance was significantly reduced in animals treated with TNFα antagonist of either kind, correlating with less macrophage accumulation around collateral arteries [34]. Inducible nitric oxide synthase (iNOS), being highly expressed by M1 macrophages as well, plays an essential role in tumor development and angiogenesis and inhibition of iNOS resulted in less collateral vessel remodeling after femoral artery occlusion [35,36]. FGF-2 also has pro-arteriogenic properties as was demonstrated by a study in rats which underwent femoral artery ligation followed by daily infusion of FGF-2 by osmotic pumps. As a result, collateral blood flow was significantly increased in the first two weeks after occlusion [37]. Furthermore, it was demonstrated that during the early phase of arteriogenesis FGFR-1 expression was increased, in particular in smooth muscle cells (SMCs), suggesting that collateral remodeling is mediated by FGFR-1 availability [38]. Since TNFα is expressed by classically activated M1 macrophages [39] and FGF-2 expression is highly increased in M2a macrophages (manuscript submitted), the observation of Arras et al suggests that M1 and M2 macrophages co-exist during the early phase of collateral remodeling and both invest in promoting collateral growth. This was confirmed by a recent study where the distribution of macrophage subsets, in both space and time, was investigated after femoral artery ligation. A strong increase in M2 macrophages was observed shortly after occlusion followed by increased numbers of M1 macrophages on day 3, after which both populations continue to increase over time. Whereas M1 macrophages were detected adjacent to the media, M2 macrophages were positioned in the outer perivascular area. Speculations about the function of this spatial distribution were made by the authors, suggesting a monocyte attracting function for the M1 subset close to the lumen and a vascular remodeling function for the M2 subset in the perivascular space [40]. Co-existence of both macrophage populations during collateral remodeling together with our current findings proposes complementary mechanisms (including TNFa and FGF-2 production by M1 and M2 macrophages respectively) of equal importance for each subtype during vascular remodeling.

In this study we showed that local infusion of polarized macrophages stimulated arteriogenesis. The pro-arteriogenic effect of macrophage subsets is most likely the result of a concerted action of macrophage-derived cytokines and growth factors, which have proven pro-angiogenic and pro-arteriogenic properties. We did not find enhancement of collateral flow by non-polarized macrophages, so the paracrine stimulation of specific cytokines and growth factors produced by polarized macrophages likely stimulates collateral formation. Polarization might lead to complementary actions of macrophages in the arteriogenic process rather than specialization of one macrophage subset.
